# The Cyclobutanocucurbit[5–8]uril Family: Electronegative Cavities in Contrast to Classical Cucurbituril while the Electropositive Outer Surface Acts as a Crystal Packing Driver

**DOI:** 10.3390/molecules26237343

**Published:** 2021-12-03

**Authors:** Minghua Chen, Naixia Lv, Weiwei Zhao, Anthony I. Day

**Affiliations:** 1 College of Biology and Chemistry, Xingyi Normal University For Nationalities, Xingyi 562400, China; gui_zhou_chen@163.com (M.C.); xiaoxia791102@163.com (N.L.); 2 Pharmaron Beijing Co., Ltd., Beijing 100176, China; kingzhao@yeah.net; 3 Chemistry, School of Science, University of New South Wales Canberra, Australian Defence Force Academy, Canberra, ACT 2600, Australia

**Keywords:** cyclobutanocucurbituril, cucurbituril, dipole interactions, electronegative cavities, molecular host

## Abstract

The structural parameters for the cyclobutanoQ[5–8] family were determined through single crystal X-ray diffraction. It was found that the electropositive cyclobutano methylene protons (CH_2_) are important in forming interlinking crystal packing arrangements driven by the dipole–dipole interactions between these protons and the portal carbonyl O of a near neighbor. This type of interaction was observed across the whole family. Electrostatic potential maps also confirmed the electropositive nature of the cyclobutano CH_2_ but, more importantly, it was established that the cavities are electronegative in contrast to classical Q[5–8], which are near neutral.

## 1. Introduction

Cucurbit[*n*]uril (classical Q[*n*], *n* = 5–8, 10, and 13–15) have been shown to exhibit a wide range of potential applications in various fields, through leveraging the properties of this macrocyclic family and by the selection of a cavity with an appropriate size. Recent examples demonstrate this with targeted cell imaging [1], human cancer assay [2], Q[*n*]-based supramolecular frameworks (QSFs) [3,4], sensors [5,6,7,8], fluorescent imprintable hydrogels [9], fluorescent probes [10,11,12], nitroxide radical probes [13], the preparation of adsorbent or solid fluorescent materials [14], room-temperature phosphorescence (RTP) [15,16], light-harvesting systems [17], nanocapsules [18], nanofiltration membranes [19] molecular machines [20,21,22] reductive catalysis of CO_2_ [23], and gold recovery [24].

**Figure 1:**
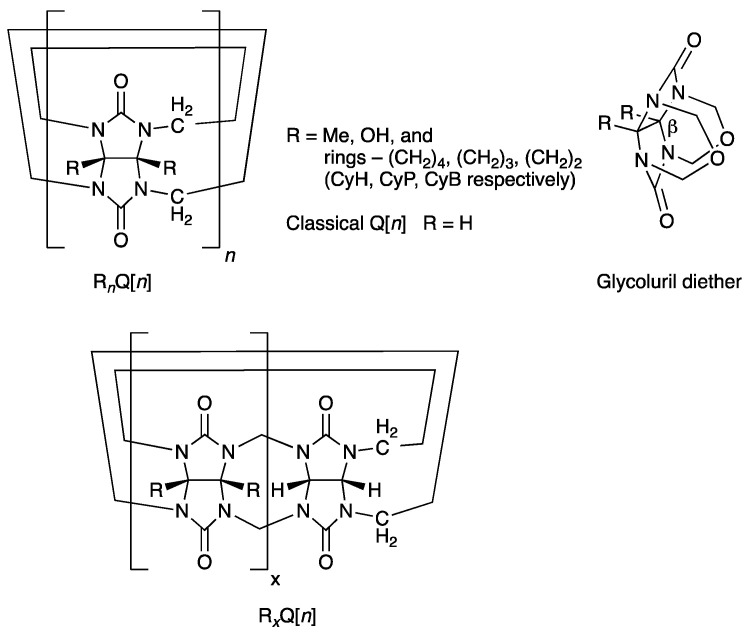
Classical Q[*n*] is represented above, where all R = H (top LHS). Fully substituted derivatives R_n_Q[*n*] are shown for the five known examples (top LHS). Partially substituted derivatives are R_x_Q[*n*] (bottom). Glycoluril diether identifying the β° angle is discussed in the text (RHS).

A bank of fully substituted derivatives of Q[*n*] have slowly emerged in the literature between 1992–2017, where the equatorial regions have been decorated with methyl (Me), hydroxyl (OH) groups, or the fused rings cyclohexano (CyH), cyclopentano (CyP), and cyclobutano (CyB) (Figure 1, R*_n_*Q[*n*]) [25,26,27,28,29,30]. The alkyl-substituted examples are all synthesized by the H^+^/cat. condensation and oligomerization of an appropriately substituted glycoluril with HCHO or from its diether alone. However, bearing Me or CyH substituents only favors the formation of the smaller homologues *n =* 5 (major) and 6 [25,26,27]. Significantly, the contracted rings, especially CyB, enable the formation of higher homologues *n =* 7 and 8 [29,30]. Just prior to our reporting of the synthesis of the fully substituted CyB_8_Q[8] the partially substituted Me_4_Q[8] and CyH_2_Q[8] were the only higher homologues (*n* = 8) carrying substitution that are prepared by condensation and cyclo-oligomerization (Figure 1, bottom R*_x_*Q[*n*]) [31].

The motivation for examining the contracted ring substituents CyP and then CyB related to a hypothesis that the dihedral angle, β°, of the fused imidazolidinone rings at the concave face was an important contributor to the formation of higher homologues. This was especially relevant to the synthesis of fully substituted higher homologues, which were otherwise unavailable. As a follow-up to the previous work, we included theoretical calculations for the angle β° for each of the precursor glycoluril diethers (R = Me, CyH, CyP, CyB, Figure 1). Using this theory, the angle for each was not only available to compare to measured values as a verification but also to provide calculated values, which would otherwise not be available.

Herein, we report a repeat of the initial synthesis of CyB_5–8_Q[5–8] with the objective of obtaining crystal structures for each of the homologues [30]. This not only provides support for the original findings but also enables the collection of important structural data to better understand possible influences on their physical and chemical properties. Particularly relevant is the diameter of the cavities and the portal openings in comparison to homologues of classical Q[*n*] and/or different substitutions.

In solution, it was previously observed that equatorial substitution has an effect upon the binding affinities of various molecular guests, where affinities can increase or decrease relative to the same guest molecule [20,21,22,29,30,31,32]. In the case of partially substituted derivatives such as Me_4_Q[6] and Me_4_Q[8] (Figure 1, bottom), a distortion in the cavity can partly explain a change in the binding affinity, which is also dependent upon the guest’s shape [20,21,22,31], whereas, with full substitution, the Q cavities are spheroidal and effects upon binding affinities in solutions can best be explained by electronic changes, the degree of Q structure rigidity, and variations in diameters of the portals and cavities. Two significant examples that demonstrate this were previously reported for comparative binding affinities for classical Q[6] and CyP_6_Q[6] (Figure 1) for the guest ions cyclohexylammonium and octane-1,8-diammonium, respectively, ~120- and 8-fold higher in the latter host [20]. The explanation for the increase is primarily related to an increase in electron density on the carbonyl O contributed by the equatorial alky substituents [21].

In addition to the physical dimensions, electronic surface effects in the solid state were reported by Tao and coworkers for classical Q[*n*] crystal packing. They found a strong interactive correlation between the electropositive outer surface of Qs with a neighboring Q electronegative portal and/or anions, in particular, the anion [ZnCl_4_]^2−^ or similar large anions [3].

The objectives in this study were to determine the physical and electronic similarities or differences between CyB_5–8_Q[5–8] and classical Q[*n*].

## 2. Results and Discussion

We were fortunate in being able to obtain single crystals of all four homologues with suitable quality for X-ray diffraction. The CyB_5_Q[5] was obtained in the first crop of crystals from acidic water (~0.05 M) as CyB_5_Q[5]·7H_2_O (**1**). The second crop of crystals from the same solution, which at this point had become more concentrated due to evaporation, afforded CyB_6_Q[6] as CyB_6_Q[6]·2Cl^−^·2(H_3_O^+^)·14H_2_O (**2**). The remaining two homologues, CyB_7_Q[7] and CyB_8_Q[8], were concentrated into product-rich fractions by silica gel chromatography of the residue obtained from the filtrate after the collection of the co-crystallized CyB_5–6_Q[5–6]. CyB_7_Q[7] was crystallized from dilute HCl as CyB_7_Q[7]·12H_2_O (**3**), while the crystallization of CyB_8_Q[8] was facilitated with the addition of ZnCl_2_ in dilute HCl to afford CyB_8_Q[8]·(ZnCl_3_·H_2_O)^−^·H_3_O^+^·10H_2_O (**4**).

The first notable feature of the family of CyB_5–8_Q[5–8] was the similarity in their dimensions to the classical Q[5–8] family. A comparison to a selection of reported guest free classical Q[5–8] showed that the average dimensions of the portals, cavities, and depths were indiscernible from those of CyB_5-8_Q[5–8] (Table 1).

Another important feature found with the CyB_5-8_Q[5–8] family in their crystal packing arrangement is the interaction of the outer equatorial surface of CyB*_n_*Q[*n*] and electronegative portal O and/or anions. This was found with the Cl^−^ anion in the case of the crystal of CyB_6_Q[6]·2Cl^−^·2(H_3_O^+^)·14H_2_O (**2**) and the [ZnCl_3_H_2_O]^−^ anion in the case of the crystal of CyB_8_Q[8]·(ZnCl_3_·H_2_O)^−^·H_3_O^+^·10H_2_O (**4**).

In this context, we calculated the ESP maps for each of the CyB*_n_*Q[*n*] homologues. Compared to similar electrostatic potential maps (ESP) for the classical Q[5–8] there were two distinct differences [3b]. The outer equatorial surface was less positive by 10–12 kcal mol^−1^ compared to ESPs for the classical Q[5–8], however, clearly sufficiently positive, favoring interactions with the electronegative C=O in the crystal packing of **1**, **2**, **3,** and **4**, with additional anion interactions specific to **2** and **4** [3a]. Of far greater significance was the inner cavity surface potential at the widest point. Here the ESP was found to be >−12.5 kcal mol^−1^ more negative, whereas classical Q[*n*] are near neutral. This was more obvious as the cavities increased in diameter from CyB_5_Q[5] to CyB_8_Q[8] where the latter had the most negative cavity surface.

Previous reported decreases in binding affinity of guests cannot, therefore, be explained based on larger portals or spacious cavities; however, the electronic differences are a likely a factor [30].

### Highlighted Structural Features for 1, 2, 3, and 4 and Their Outer Surface Interactions

**Figure 2:**
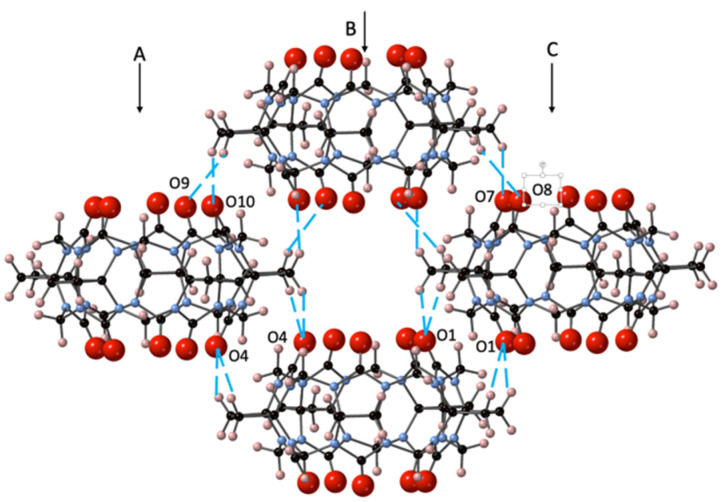
The crystal packing of CyB_5_Q[5]·7H_2_O (**1**) with the dipole–dipole interactions between portal C=O and cyclobutano CH_2_ protons indicated (– –) for a set of 4 CyB_5_Q[5] representing a segment of columns A, B, and C. The H_2_O was omitted for clarity.

The solid-state structure of CyB_5_Q[5]·7H_2_O (**1**) was found to be a relatively simple set of stacked cages forming columns side by side as a continuous corrugated sheet (Figure S1). The portals are superimposed upon the CyB_5_Q[5] below but cages of each column are out of register with cages in the adjacent column (A and B, B and C, Figure 2), creating a tube of cavities.

The asymmetric unit structure CyB_5_Q[5]·7H_2_O contains a water molecule in each portal (O1W and O2W) but none in the cavity (Figure S2a). The remaining 5H_2_O completes a H-bonding network that helps to link each CyB_5_Q[5] cage (Figure S2b and Table S1). However, the most significant driving force in the crystal packing arises through dipole–dipole interaction between the portal C=O and the cyclobutano CH_2_ protons. The lacing together of columns A, B, and C is affected with 16 associated interactions, as shown for set of four CyB_5_Q[5] (Figure 2). The specific proton connections are O1–H4A and H3B, O4–H27B and H28A, O7–H4B, O8–H3A, O9–H28B, and O10–H27A (distances 2.56, 2.54, 2.59, 2.51, 2.38, 2.81, 2.84, and 2.37 Å, respectively). Hence, there are significant dipole–dipole interactions between the electronegative C=O and electropositive cyclobutano CH_2_.

**Figure 3:**
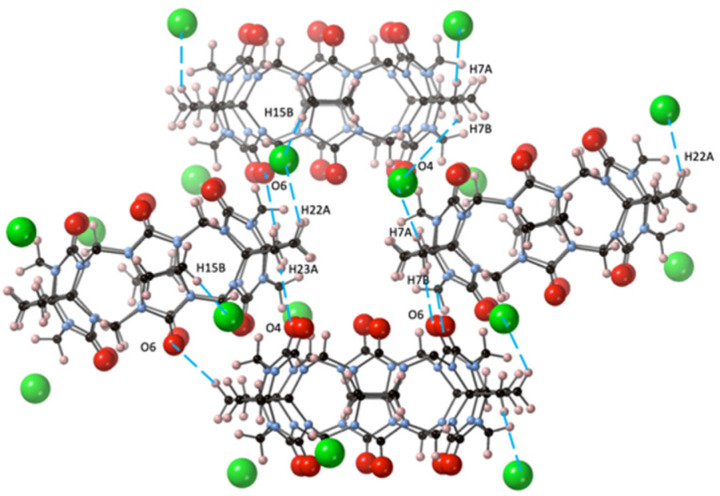
In structure **2**, the CyB_6_Q[6] outer surface dipole connections (– –) are shown between C=O and cyclobutano CH_2_ and these same protons and the Cl^−^ ion (green).

The primary driving force for the crystal packing of CyB_6_Q[6] in the crystal CyB_6_Q[6].2Cl^−^·2(H_3_O^+^)·14H_2_O (**2**) obtain from dilute HCl was also strongly influenced by the outer surface interaction of the partially positive CH_2_ protons of the cyclobutano substituent. This is reflected in the short distances between O6–H7B, O4–H23A, and Cl1–H7A (2.44, 2.75, and 2.74 Å, respectively). Slightly longer interactions between Cl1–H22A and H15 (2.94 and 3.00 Å) were also found. The portals O4 and O6 are directly connected to H23A and H7B of their cyclobutano nearest CyB_6_Q[6] neighbor (Figure 3).

**Figure 4:**
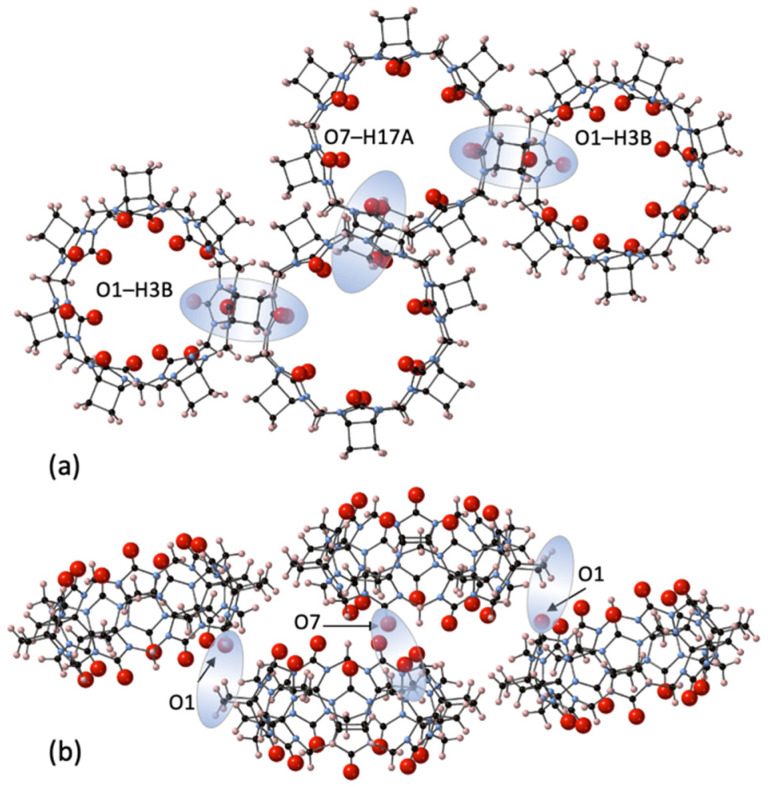
A segment of the crystal structure **3** showing the inter-column connections that are the predominant structural features as seen from (**a**) the portal view and (**b**) from the side view_,_ of the CyB_7_Q[7] stacked columns, showing the dipole–dipole interactions between O1–H3B and O7–H17A (shaded highlights).

The third largest homologue CyB_7_Q[7] was concentrated through chromatography and crystallized from dilute HCl solutions with very slow evaporation to afford CyB_7_Q[7]·12H_2_O (**3**) (Figure 4). Interestingly, no Cl^−^ ions were retained in these structures. The CyB_7_Q[7] are superimposed in closely stacked arrangements of columns, with significant quantities of water molecules congregated in line with and in close proximity to their portals. The interstitial portal spaces between each CyB_7_Q[7] had four water molecules sandwiched in these locations. One space was occupied by O3W, O4W, and O5W with O4W duplicated and O6W, O7W, and O8W occupying the opposite space with O7W duplicated (Figure S4). Surprisingly, no water molecules were found in the cavity, only just inside the portal. The remaining water O1W and O2W duplicated and sat toward the edges of two shared portals. The prime dipole driving force for close-packed column formation appeared to be the connections between the C=Os and the protons of cyclobutano CH_2_ of the closest CyB_7_Q[7] in a neighboring column (O1–H3B and O7–H17A, 2.72 and 2.94 Å, respectively).

**Figure 5:**
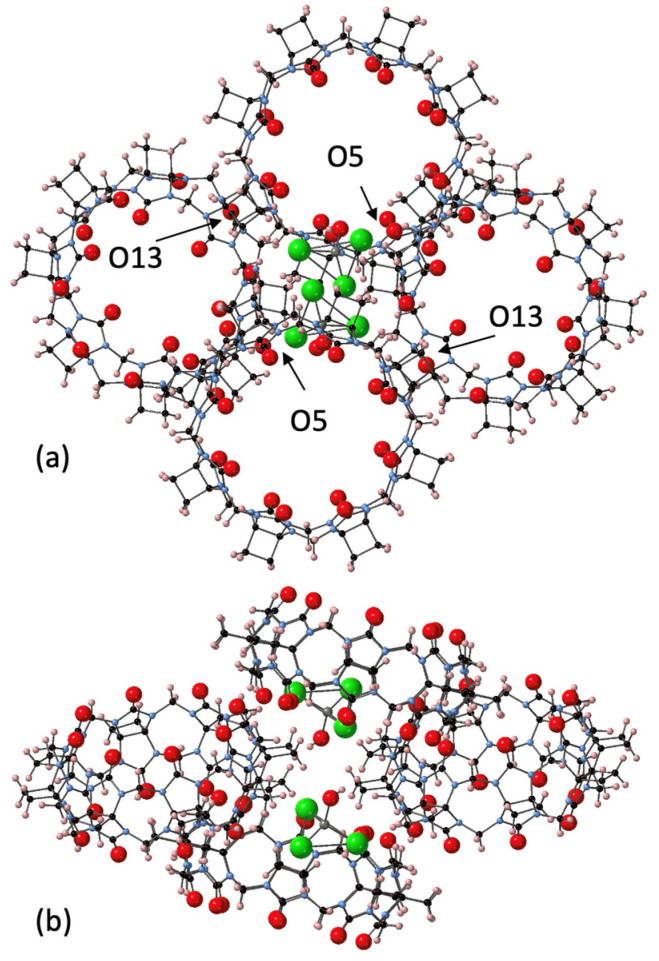
Views of the crystal packing of **4**. The close association of the cyclobutano CH_2_ outer surface of CyB_8_Q[8] with the [ZnCl_3_H_2_O]^−^ anion is shown. (**a**) O5 and O13 are labelled and have close interactions with cyclobutano CH_2_. (**b**) The same structural unit with a side view after rotating vertically 90°. All water molecules were omitted for clarity, except Zn-coordinated H_2_O.

The largest homologue CyB_8_Q[8] was successfully crystallized with the assistance of a chlorozincate anion, which resulted in the crystals CyB_8_Q[8]·(ZnCl_3_·H_2_O)^−^·H_3_O^+^·10H_2_O (**4**). The CyB_8_Q[8] cages were found to be knitted together by the [ZnCl_3_H_2_O]^−^ anion into an apparent honeycomb structure (Figure 5a). Multiple close associations with the [ZnCl_3_H_2_O]^−^ anion and the electropositive cyclobutano protons with contact distances of ~ 2.8 Å created a cluster of 3xCyB_8_Q[8]s around a single anion (Figure 5b). The second [ZnCl_3_H_2_O]^−^ anion shown and the fourth CyB_8_Q[8] was the beginning to the next stacked layer. The porosity of the solid-state structure was obvious with the omission of H_2_O. In addition, the portals and cavities were not obstructed by anions (Figure 5a). H_2_O was found H-bonded to the C=O just inside the portals, close to the Cl of the anion and between the outer surfaces of the CyB_8_Q[8] (Figure S5). The primary driving force for packing appeared to be the ion–dipole interaction between electropositive protons on the outer surface and the anion. However, close portal C=O to cyclopentano CH_2_ interactions were found between O5–H60A, O13–H5B, and a slightly longer interaction O15–H4A, 2.74, 2.64, and 2.98 Å, respectively.

Collectively, each homologue was found to have significant outer surface interactions between the electropositive cyclobutano CH_2_ and the electronegative portal C=O and/or the anions Cl^−^ or [ZnCl_3_H_2_O]^−^ that contribute to the crystal packing of each structure. The directionality of the CH_2_ relative to the cavities favors the formation of near-parallel cavity columns, which contrasts with the classical Q[*n*]. The equatorial protons of classical Q[*n*] protrude at 90° relative to the cavity axis and, therefore, allow direct portal interaction with a near neighbor, leading to orientation of cavities perpendicular to each other.

**Figure 6:**
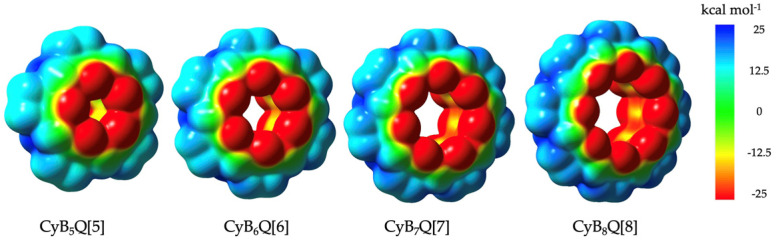
Electrostatic potential maps (ESPs) for CyB_5_Q[5], CyB_6_Q[6], CyB_7_Q[7], and CyB_8_Q[8], respectively.

The electropositive outer surface of the family of CyB_5–8_Q[5–8] was supported by ESP calculations (Figure 6). However, compared to the classical Q[5–8], two distinct differences were found, which are highlighted in Table 2 [4]. The first was that the outer equatorial surface was less positive by 10–12 kcal mol^−1^ compared to ESPs for the classical Q[5–8] but clearly sufficiently positive to favor interactions with the electronegative C=O in the crystal packing of **1**, **2**, **3,** and **4** with additional anion interactions specific to **2** and **4** [3a]. The second, but more important, difference is at the inner cavity surface at the widest point. Here the ESP > −12.5 kcal mol^−1^ was more negative, whereas classical Q[*n*] were near to neutral. The visible color change of the ESP map of the cavity surfaces from yellow to nearly all red was obvious as the cavities increased in diameter, with CyB_8_Q[8] being the most negative (Figure 6).

A feature not discussed so far that could impact the size of the cavities of CyB_5–8_Q[8] is the β° angle of the cis-fused imidazolidinone rings of the glycoluril moieties. As an average this was found to be ~0.8° wider than classical Q[5–8]. As a poignant comparison, the six smaller homologues of Me_10–12_Q[5–6], classical Q[5–6], and CyB_5–6_Q[5–6] (Figure 1 R = Me, H and CyB, respectively) each had differences, with the most striking occurring between the Me-substituted examples compared to the other two types (β = 112.17 and 112.84° for Me substitution, respectively, β > 3° wider angle, Table 3). This difference occurred as a function of the substituent carried at the cis-fused junction of the glycoluril moiety.

The findings above underline the significance of the β° angle of the glycoluril precursors (such as the diethers, Figure 1 RHS) to the synthesis of fully substituted higher homologues.

In addition to ESP theory, we also calculated the dihedral β° of the precursor glycoluril diethers R = Me, CyH, CyP, and CyB and where R = H, the latter an unknown compound (Figure 1). The interest here was 2-fold: to determine through calculation the angle β° for the diethers including R = H, which has no measurable data available, and, secondly, to compare theoretical values with those previously measured. A good fit of theory to measure would also support future applications as a predictive tool to identify suitable glycoluril candidates for the synthesis of higher homologues. The theoretical values compared well for the three cyclo-substituted examples and R = Me, as shown in Table 4. However, R = CyB and Me were slightly over estimated.

The calculated trend toward a wider angle from CyH to CyB (6–4 membered ring substituents) and a consistency with the measured angle is encouraging and potentially could be applied to future theoretical glycoluril diethers and ultimately to the synthesis of newly substituted Q[*n*] families. The measured trend is also consistent in Q[*n*] derivatives, although the angles are wider due the involvement of eight-membered rings joining neighboring glycoluril moieties as opposed to six-membered rings for the glycoluril diethers.

## 3. Materials and Methods

Starting materials were purchased from commercial suppliers and used without further purification. CyB_5-8_Q[5–8] were prepared in accordance with the literature method [30]. NMR spectra were identical to those previously reported.

### 3.1. Purification of CyB_5_Q[5]·7H_2_O *(**1**)* and CyB_6_Q[6]·2Cl^−^·2(H_3_O^+^)·14H_2_O *(**2**)*

The crude mixture of CyB*_n_*Q[*n*] was obtained as a solid (2.76 g), which also contained LiCl (0.1 g) as part of the reaction process. Distilled water (50 mL) was added, and the mixture was heated to dissolve the bulk of the material. The undissolved material (1.15 g) was collected by filtration and the filtrate was set aside at RT, over a period of days, which resulted in successive quantities of precipitate, also collected by filtration (0.81 g). The undissolved material and the collected precipitates were combined. The total of the collected solids (1.96 g) were then completely dissolved in distilled water (30 mL) following the addition of HCl 32% (8–10 drops). After 30 days at RT, crystals of **1** were obtained (0.15 g). The filtrate was then set aside at RT for 40 days, which yielded crystals of **2** (0.08 g).

### 3.2. Purification of CyB_7_Q[7]·12H_2_O *(**3**)* and CyB_8_Q[8]·(ZnCl_3_·H_2_O)^−^·H_3_O^+^·10H_2_O *(**4**)*

The filtrate above, obtained from the solution remaining after the collection of the co-crystallized CyB_5–6_Q[5–6], was evaporated to dryness and the residue (0.85 g) was subjected to silica gel column chromatography eluting with a mixture of HCO_2_H/AcOH/EtOH (1:5:0.1). After the early fractions were clear of remaining CyB_5–6_Q[5–6] (as determined by tlc), the eluant was changed to HCO_2_H/AcOH (1:2). Fractions of predominantly CyB_7_Q[7] were combined and the solvent was evaporated in vacuo. Modification of the eluant ratio to 1:1 gave CyB_8_Q[8]-rich fractions, which were also combined, and the solvent was evaporated.

The solid residue from the CyB_7_Q[7]-rich fractions were dissolved in diluted HCl 0.03 M (2 mL) and set aside for ~50 days with slow evaporation to obtain single crystals of **3**. The residue from the CyB_8_Q[8]-rich fractions were also dissolved in diluted HCl 0.03 M (2 mL) with added ZnCl_2_ (0.3 g). After a similar time period with slow evaporation, single crystals of **4** were obtained.

### 3.3. X-ray Crystallography

X-ray crystal data for complexes **1**–**4** were collected on a Rigaku Oxford Diffraction Supernova Dual Source (Oxford Diffraction Ltd., Abingdon, England) Cu at zero equipped with an AtlasS2 CCD using Mo-Kα radiation. Lorentz polarization and absorption corrections were applied. Structural solutions and full-matrix least-squares refinements based on F^2^ were performed using the SHELXT-14 and SHELXL-14 program packages, respectively. All non-hydrogen atoms were refined anisotropic thermal parameters. Analytical expressions of the neutral atom-scattering factors were employed and anomalous dispersion corrections were incorporated. A summary of the crystallographic data, collection conditions, and refinement parameters for complexes **1**–**4** are listed in Table 5. Crystallographic data (excluding structure factors) for the structure reported in this paper have been deposited with the Cambridge Crystallographic Data Centre as deposition Nos. CCDC 1997294, 2050695, 2050988, and 20501000. Copies of the data can be obtained free of charge on application to CCDC, 12 Union Road, Cambridge CB2 1EZ, UK (Fax: +44 1223/336 033; e-mail: deposit@ccdc.cam.ac.uk).

### 3.4. Theoretical Method

The theoretically calculated dihedral angle β° of the fused imidazolidinone rings at the concave face for each glycoluril diether and the ESP was determined for CyB_5–8_Q[5–8] using the B3LYP/6-311G (d, p) level of theory from Gaussian 09 calculations.

## 4. Conclusions

The purification and separation of substituted Q homologues is always a challenge and here we were able to separate the two more difficult larger homologues on silica gel using polar solvent gradients.

The structural parameters for the family of CyB_5–8_Q[5–8] are similar to classical Q[5–8] in their portal and cavity dimensions. Electronically, however, significant electronegative potentials were found within the cavities of CyB_5–8_Q[5–8], unlike the classical Q[*n*], which are near neutral based upon comparative ESPs. The outer equatorial surfaces of CyB_5–8_Q[5–8] are relatively positive but not to the same extent as the classical Q[*n*]. The electropositive nature of the cyclobutano CH_2_ plays an important role in the crystal packing of each of the four homologues through dipole–dipole interactions. As the electropositive CH_2_ are nearly aligned with the cavity axes, packing for all four homologues formed parallel or near-parallel cavity columns.

Given that the physical dimensions are similar to the classical Q[*n*], but that the cavity electrostatic potentials are negative, this latter difference could be significant with regard to decreases in the guest binding constants mentioned in the introduction.

## Figures and Tables

**Table 1:** A comparison of the dimensions of CyB5–8Q[5–8] to classical Q[5–8] derived from X-ray crystal structures.

Q[*n*]	Portal O–O Average Diam Å	Cavity C–C Average Diam Å	Depth Å
Q[5] CyB_5_Q[5]	2.11 2.15	5.22 5.22	9.13 [33] 9.13
Q[6]Na CyB_6_Q[6]	3.80 3.96	6.80 6.77	9.08 [34] 9.15
Q[7] CyB_7_Q[7]	5.17 5.21	8.20 8.22	9.09 [33] 9.13
Q[8] CyB_8_Q[8]	6.91 6.99	9.79 9.76	9.13 [35] 9.16

Dimensions include van der Waals radii.

**Table 2:** Relative ESP mapped energies (kcal mole^−1^) of classical Q[*n*] and CyB*_n_*Q[*n*] comparing the surfaces of their cavities, equatorial regions, and portals.

	Cavity C–C	Equatorial CH/CH2	Portal C=O
Q[*n*]	0 ± 5	+25	−25 [4]
CyB*_n_Q*[*n*]	−12.5	+12.5	−25

**Table 3:** A comparison of the β angle of the cis-fused junction of the glycoluril moieties of classical Q[*n*], Me*_2n_*Q[*n*], and CyB*_n_*Q[*n*]), with the difference shown as ∆°.

	Average β°		(CyB*_n_*Q[*n*]–Q[*n*])	(CyB*_n_*Q[*n*]–Me*_2n_*Q[*n*]) ∆°
	N–C–N		∆°	
Me_10_Q[5]	112.17	[36] [33]		
Q[5]	115.14			
CyB_5_Q[5]	115.98		0.84	3.81
Me_12_Q[6]	112.84	[37] [34]		
Q[6]	114.63			
CyB_6_Q[6]	115.83		1.2	2.99
Q[7]	114.65	[33]		
CyB_7_Q[7]	115.2		0.55	- ^1^
Q[8]	114.51	[35]		
CyB_8_Q[8]	115.21		0.7	- ^1^

^1^ The synthesis of Me_2n_Q[*n*] *n* = 7 or 8 is not known.

**Table 4:** A comparison of calculated and measured dihedral angles β° for the different substituted glycoluril diethers shown in Figure 1.

Substituent R =	Calculated β°	Measured β° [29,30]
H	111.9	-
CyB	112.8	111.77
CyP	110.5	110.08
CyH	109.8	109.31
Me	110.2	108.88

**Table 5:** Summary of single crystal X-ray diffraction results for compounds **1–4**.

	1	2	3	4
Formula	C_40_H_54_N_20_O_17_	C_48_H_82_Cl_2_N_24_O_28_	C_56_H_80_N_28_O_26_	C_64_H_89_Cl_3_N_32_-O_28_Zn
FW	1087.03	1514.27	1561.48	1926.39
T/K	100	293	293	110
Crystal system	triclinic	monoclinic	orthorhombic	triclinic
Space group	P-1	P1 2_1_/c 1	P nma	P-1
a [Å]	12.3691(7)	11.3747(6)	13.4181(8)	15.8972(12)
b [Å]	14.8265(17)	17.0087(7)	23.0795(18)	18.3412(14)
c [Å]	17.6974(10)	17.4352(7)	28.3746(19)	20.3221(14)
α [°]	75.361(7)	90	90	78.560(6)
β [°]	89.902(5)	93.656(4)	90	75.665(6)
γ [°]	74.370(7)	90	90	70.009(7)
V [Å^3^]	3016.4(4)	3366.3(3)	8787.1(10)	5352.8(7)
Z	2	2	4	2
R [I > 2σ(I)] ^1^	0.1181	0.0877	0.0954	0.0912
wR [I > 2σ(I)] ^2^	0.3249	0.2500	0.2575	0.2256

^1^ Conventional R on Fhkl: ∑||F_o_| − |F_c_||/∑|F_o_|. ^2^ Weighted R on |Fhkl|^2^: ∑[w(Fo^2^ − Fc^2^)^2^]/∑[w(Fo^2^)^2^]^1/2^.

## Data Availability

The data is available in a publicly accessible repository.

## Supplementary Materials

The following are available online. Figures S1 and S2: Crystal packing of CyB_5_Q[5]·7H_2_O (1) including and excluding structural water. Figure S3: Crystal packing of CyB_6_Q[6]·2Cl^−^·2(H_3_O^+^)·14H_2_O (2) including structural water. Figures S4 and S5: Crystal packing of CyB_7_Q[7]·12H_2_O (3) including structural water. Figure S6: Crystal packing of CyB_8_Q[8]·(ZnCl_3_·H_2_O)^−^·H_3_O^+^·10H_2_O (4) including structural water. Table S1: The water H-bond parameters of complexes 1–4.

## Sample Availability

Samples of the compounds are available from the authors.
