# Supplementary material for: The Cyclobutanocucurbit[5–8]uril Family: Electronegative Cavities in Contrast to Classical Cucurbituril while the Electropositive Outer Surface Acts as a Crystal Packing Driver
Source: Molecules. 2021 Dec 3;26(23):7343. doi: 10.3390/molecules26237343 (PMC8659056; doi:10.3390/molecules26237343)
Supplement: Supplementary file 1 [file molecules-26-07343-s001.zip › molecules-1457022-supplementary.pdf]

Supporting Information S1

The Cyclobutanocucurbit[5–8]uril Family: Electronegative Cavities in Contrast to Classical Cucurbituril while the Electropositive Outer Surface Acts as a Crystal Packing Driver

Minghua Chen,[1] Naixia Lv,[1] Weiwei Zhao[2] and Anthony I. Day[3,*]

Table of Contents:

	Page
Figure S1 and S2 Crystal packing of $CyB_5Q[5]\cdot 7H_2O$ (1) including and excluding structural water	**S2**
Figure S3 Crystal packing of Crystal packing of $CyB_6Q[6]\cdot 2Cl^-\cdot 2(H_3O^+)\cdot 14H_2O$ (2) including structural water	**S3**
Figure S4 and S5 Crystal packing of $CyB_7Q[7]\cdot 12H_2O$ (3) including structural water	**S4**
Figure S6 Crystal packing of $CyB_8Q[8]\cdot (ZnCl_3\cdot H_2O)^-\cdot H_3O^+\cdot 10H_2O$ (4) including structural water	**S5**
Table S1 The water H-bond parameters of complexes 1-4	**S6-7**

1.1 Crystal packing of $CyB_5Q[5]\cdot 7H_2O$ (1)

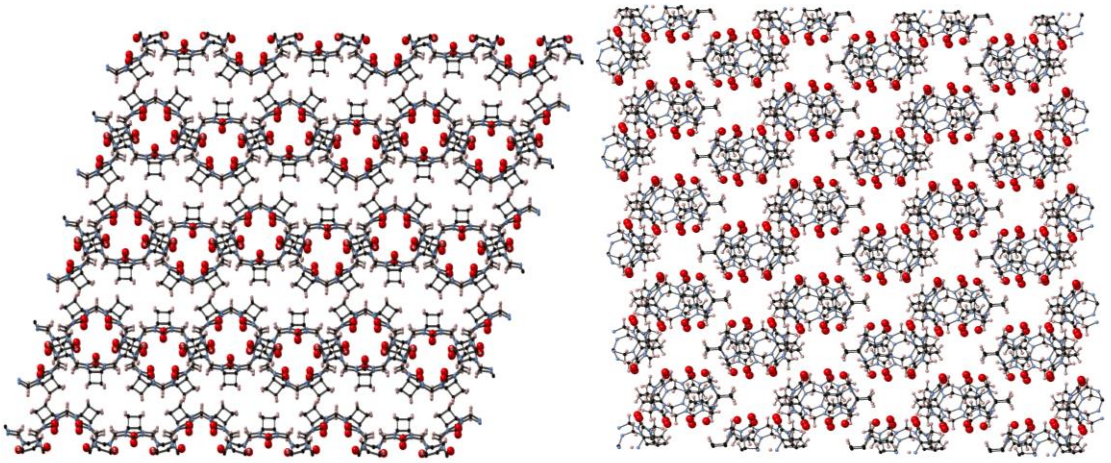

Figure S1. LHS $CyB_5Q[5]$ crystal packing viewed through the portals showing the formation of corrugated sheets. The free H_2O has been omitted which lies between the sheets. RHS is the side view of the $CyB_5Q[5]$ aligned in columns but out of register with their neighbouring column (x-y face) H_2O omitted.

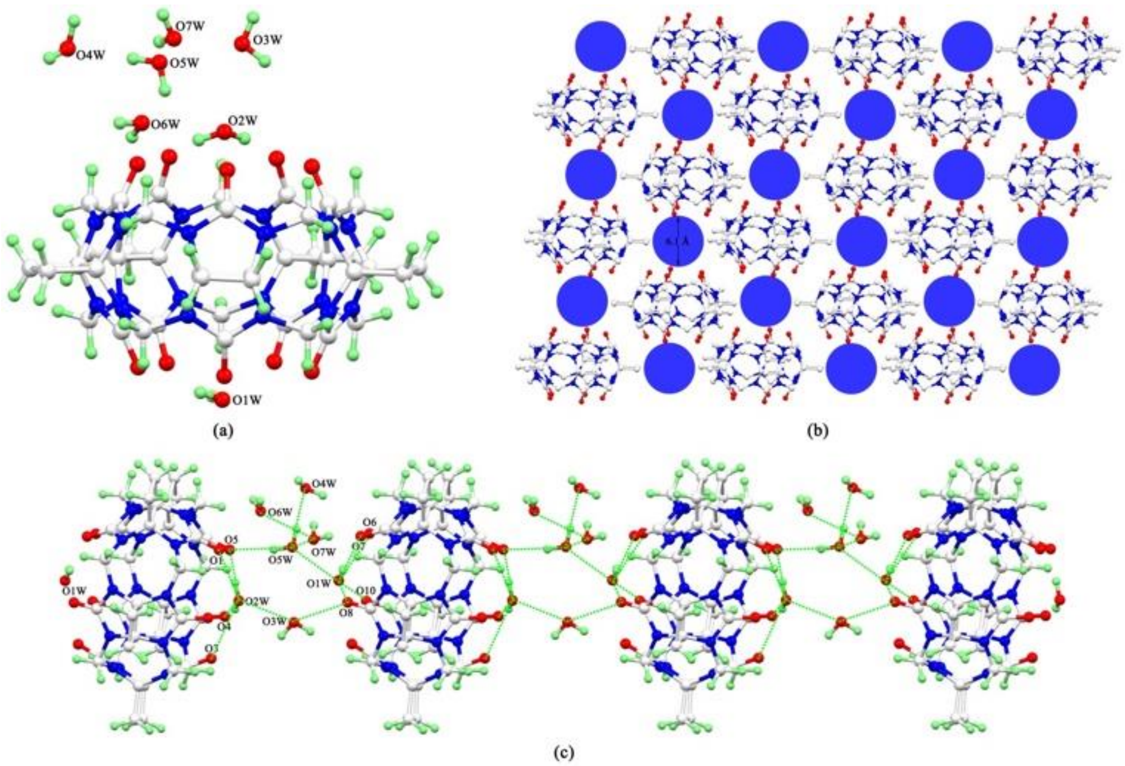

Figure S2. Crystal structure of $CyB_5Q[5]\bullet 7H_2O$ ： (a) Asymmetric unit, (b) 3-D stacking with water channels blue discs (~6.1 Å), (c) H-bonding interactions with free H_2O and 1-D supramolecular chain of $(CyB_5Q[5]\bullet 7H_2O)n$.

1.2 Crystal packing of $CyB_6Q[6]\cdot 2Cl^{-}\cdot 2(H_3O^{+})\cdot 14H_2O$ (2)

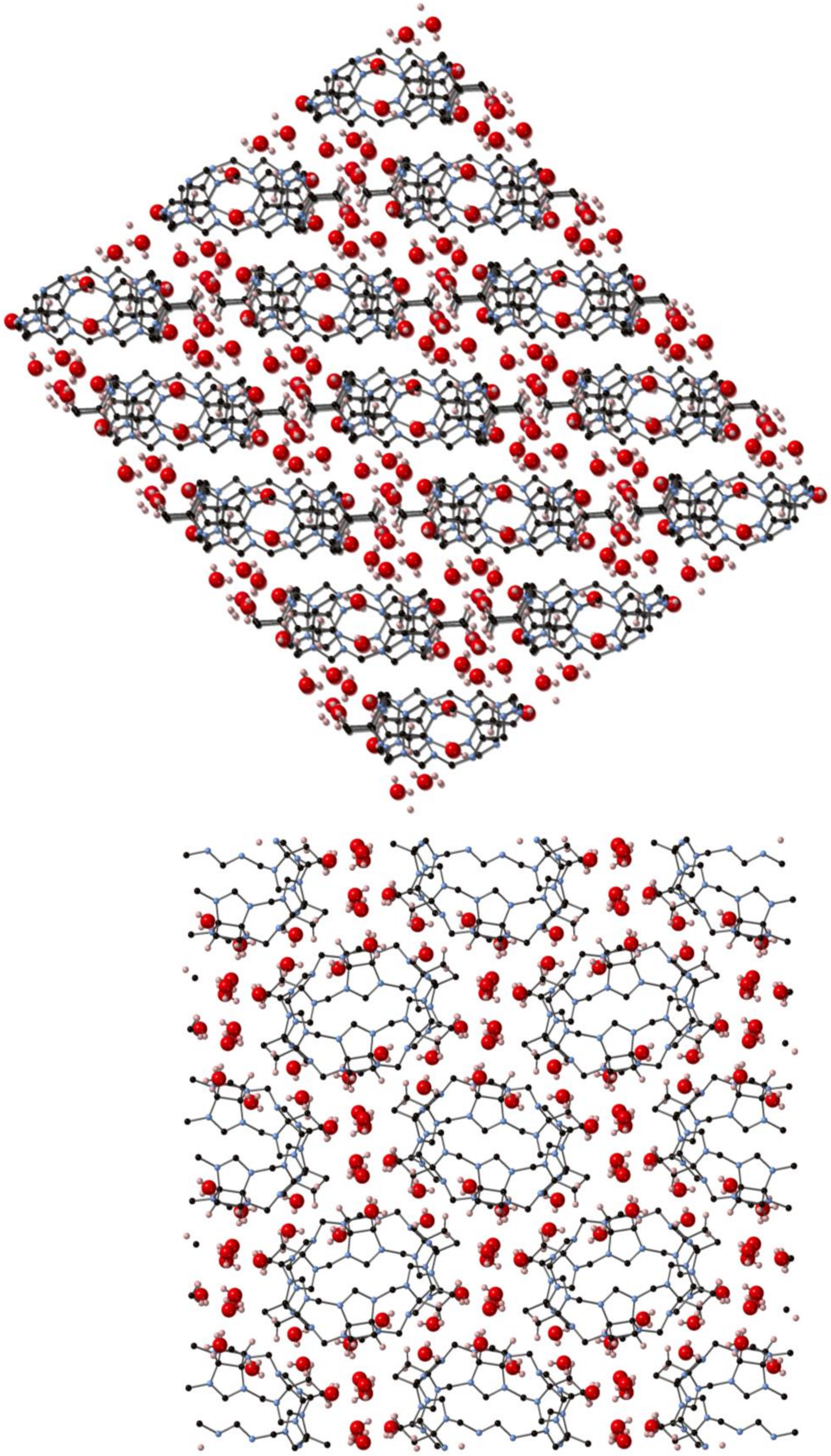

y-z face

Figure S3. All free water has been included and all carbonyl O and chloride atoms have been omitted for clarity. (Top) x-z face gives a side view of the $CyB_6Q[6]$ cages free water location. (Bottom) y-z face a slightly tilted portal view showing free water locations.

1.3 Crystal packing of $CyB_7Q[7]\cdot 12H_2O$ (3)

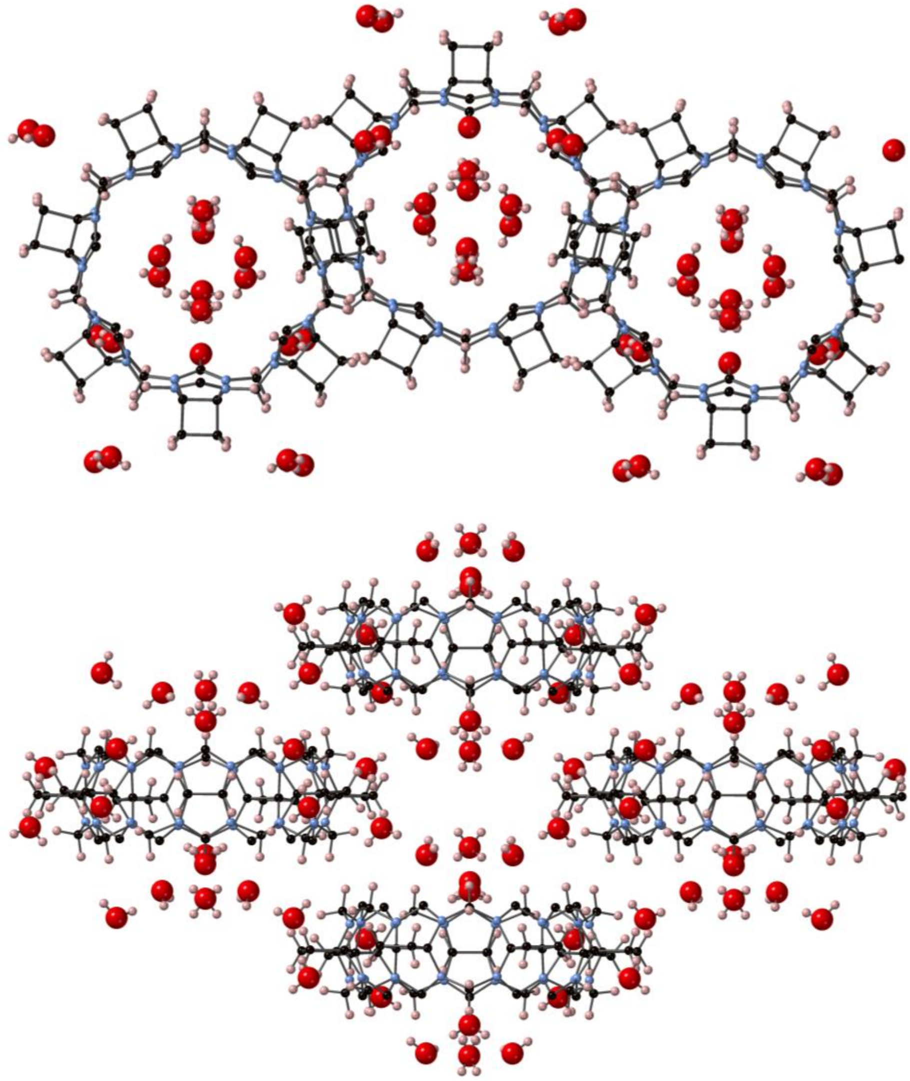

Figure S4. All free water has been included and all carbonyl O atoms have been omitted for clarity. (Top) Portal view

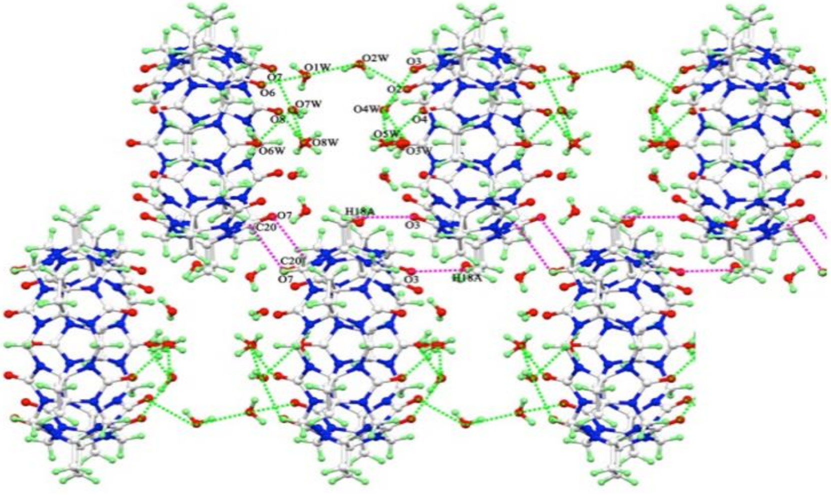

Figure S5. Showing the H-bonding network

1.4 $CyB_8Q[8]\cdot(ZnCl_3\cdot H_2O)^{-}\cdot H_3O^{+}\cdot 10H_2O$ (4)

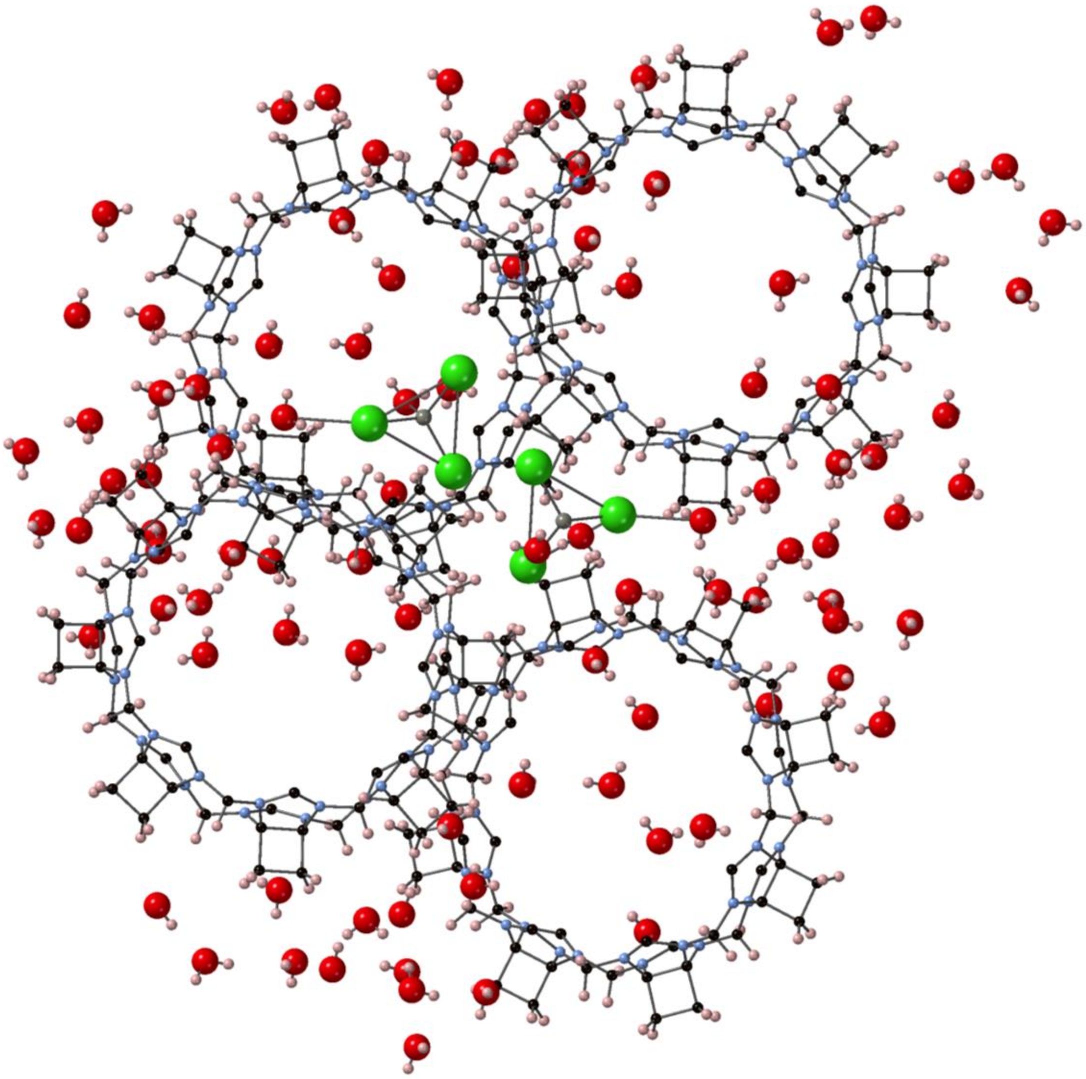

Figure S6. All free water has been included and all carbonyl O atoms have been omitted for clarity.

Table S**1**. The water H-bond parameters of complexes 1-4.

complexes	bond	bond length/Å			bond angle/°
	X—H...Y	X—H	H...Y	X...Y	X—H...Y
1	O2W—H2WA...O1	0.850	2.246	2.874	130.68
	O2W—H2WB...O3	0.850	2.546	2.898	106.01
	O2W—H2WB...O4	0.850	2.061	2.885	163.07
	O2W—H2WA...O5	0.850	2.211	2.888	136.47
	O1W—H1WA...O6	0.850	2.140	2.900	148.67
	O1W—H1WB...O7	0.849	2.049	2.848	156.69
	O1W—H1WB...O8	0.849	2.540	2.975	112.92
	O1W—H1WA...O10	0.850	2.459	2.809	105.56
	O3W—H3WA...O2W	0.849	2.578	2.804	96.50
	O3W—H3WB...O8	0.850	2.533	2.956	111.79
	O5W—H3WA...O4W	0.850	1.967	2.778	158.94
	O5W—H3WB...O5	0.850	1.992	2.837	172.91
	O7W—H7WA...O6	0.849	2.481	2.733	98.05
2	O1W—H1WA...O4	0.850	2.352	2.984	131.51
	O1W—H1WB...O5W	0.851	1.933	2.758	163.13
	O6W—H6WB...O1	0.850	2.492	2.802	102.47
	O2W—H2WA...O5	0.850	2.149	2.896	146.62
	O3W—H3WA...O2W	0.849	2.006	2.651	132.11
	O4W—H4WA...O3W	0.851	2.145	2.816	135.52
	O6W—H6WA...O4W	0.850	2.137	2.736	127.13
	O7W—H7WB...O3	0.849	2.117	2.807	138.16
	O8W—H8WB…O1	0.849	2.I25	2.784	134.12
	O4W—H4WB…Cl1	0.849	2.403	2.903	118.18
	O3W—H3WB…Cl1	0.851	2.784	2.960	93.44
	O5W—H5WB…Cl1	0.850	2.460	3.223	149.62
3	O2W—H2WB...O2	0.849	1.979	2.739	148.39
	O2W—H2WA...O3	0.851	1.879	2.689	158.68
	O1W—H1WA...O6	0.851	2.342	2.844	118.15
	O7W—H7WA...O8W	0.850	1.989	2.743	147.37
	O4W—H4WA...O3W	0.848	2.057	2.775	142.10
	O8W—H8WA...O8	0.850	2.047	2.897	178.05

	O5W—H5WB...O4	0.846	2.287	3.017	144.57
4	O1W—H1WA...O2W	0.851	2.363	3.029	135.43
	O3W—H3WC...O2W	0.848	2.175	3.004	165.85
	O3W—H3WB...O4W	0.850	1.946	2.780	166.87
	O4W—H4WB...O5W	0.849	2.363	2.878	119.59
	O5W—H5WA...O1	0.850	2.138	2.958	162.22
	O5W—H5WB...O3	0.849	2.710	2.863	91.64
	O6W—H6WB...O7W	0.850	1.972	2.794	152.06
	O6W—H6WA...O4W	0.850	1.960	2.741	162.17
	O7W—H7WB...O9	0.849	2.099	2.834	144.67
	O8W—H8WB...O13	0.851	2.109	2.883	150.99
	O9W—H9WA...O4	0.849	2.468	3.121	134.34
	O10W—H10WB...O9W	0.851	2.075	2.750	135.79
	O11W—H11A...O7	0.849	2.087	2.868	152.63
	O12W—H11C...O11W	0.870	1.800	2.612	154.56
	O12W—H11D...O10W	0.871	1.786	2.650	171.05
